# Benchmarking genetic interaction scoring methods for identifying synthetic lethality from combinatorial CRISPR screens

**DOI:** 10.1093/nargab/lqaf129

**Published:** 2025-09-26

**Authors:** Hamda Ajmal, Sutanu Nandi, Narod Kebabci, Colm J Ryan

**Affiliations:** Conway Institute of Biomolecular and Biomedical Research, University College Dublin, Dublin, D04 V1W8, Ireland; School of Computer Science, University College Dublin, Dublin, D04 V1W8, Ireland; School of Medicine, University College Dublin, Dublin, D04 V1W8, Ireland; Conway Institute of Biomolecular and Biomedical Research, University College Dublin, Dublin, D04 V1W8, Ireland; School of Computer Science, University College Dublin, Dublin, D04 V1W8, Ireland; School of Medicine, University College Dublin, Dublin, D04 V1W8, Ireland; Systems Biology Ireland, University College Dublin, Dublin, D04 V1W8, Ireland; Conway Institute of Biomolecular and Biomedical Research, University College Dublin, Dublin, D04 V1W8, Ireland; School of Computer Science, University College Dublin, Dublin, D04 V1W8, Ireland; The Research Ireland Centre for Research Training in Genomics Data Science, University College Dublin, Dublin, D04 V1W8, Ireland; Conway Institute of Biomolecular and Biomedical Research, University College Dublin, Dublin, D04 V1W8, Ireland; School of Computer Science, University College Dublin, Dublin, D04 V1W8, Ireland; School of Medicine, University College Dublin, Dublin, D04 V1W8, Ireland; Systems Biology Ireland, University College Dublin, Dublin, D04 V1W8, Ireland

## Abstract

Synthetic lethality (SL) is an extreme form of negative genetic interaction, where simultaneous disruption of two non-essential genes causes cell death. SL can be exploited to develop cancer therapies that target tumour cells with specific mutations, potentially limiting toxicity. Pooled combinatorial CRISPR screens, where two genes are simultaneously perturbed and the resulting impacts on fitness estimated, are now widely used for the identification of SL targets in cancer. Various scoring methods have been developed to infer SL genetic interactions from these screens, but there has been no systematic comparison of these approaches. Here, we performed a comprehensive analysis of five scoring methods for SL detection using five combinatorial CRISPR datasets. We assessed the performance of each algorithm on each screen dataset using two different benchmarks of paralog SL. We find that no single method performs best across all screens but identify two methods that perform well across most datasets. Of these two scores, Gemini-Sensitive has an available R package that can be applied to most screen designs, making it a reasonable first choice.

## Introduction

Synthetic lethality (SL) is a phenomenon whereby the simultaneous perturbation of a pair of non-essential genes results in cell death [1]. Since gene loss, via loss-of-function mutation or deletion, is frequently observed in tumour cells, SL can be leveraged to selectively target these cells, thereby minimizing toxicity to healthy cells. In recent years, extensive research has focused on exploiting SL to develop new cancer therapies [2, 3]. A significant milestone in this field was achieved with the approval of the first SL-based therapy—using PARP1 inhibitors for treating select breast, ovarian, and prostate cancer patients whose tumours have *BRCA1* or *BRCA2* mutations [2, 4].

CRISPR–Cas (clustered regularly interspaced short palindromic repeats–CRISPR-associated protein) screening technology is widely used for genome editing [5] and its advent sparked a renewed interest in finding SL in cancer [6]. It uses a guide RNA (gRNA) to identify a target DNA sequence and direct the Cas nuclease to it, where it induces double-strand break and causes disruption [5]. CRISPR double knock-out (CDKO) experiments allow us to examine the impact of simultaneously knocking out pairs of genes to study genetic interactions (GIs) between them. They have been widely used to identify synthetic lethal interactions, especially between duplicate genes (paralogs) [1, 7–11]. Typically, CDKO libraries contain paired single gRNAs (sgRNAs) knocking out two genes at once. These libraries also include sgRNAs paired with a non-targeting control sgRNA to assess the effects of knocking out each single gene independently. Some experiments may also include two non-targeting paired sgRNAs as a baseline control. gRNA abundance is measured through high-throughput sequencing at an initial time point and again at later time point(s) following lentiviral transduction.

Analysing and interpreting CDKO screen data remains challenging due to significant variations in guide activity, high replicate variability, and differences in sgRNA library design [12]. Variations in sgRNA libraries, such as the number of distinct sgRNA pairs per gene pair, the number of individual or paired non-targeting control sgRNAs, the number of sgRNAs targeting positive control genes, and the orientation of targeted gene pairs, make it difficult to apply statistical methods consistently across studies without substantial modifications or adaptations. Several statistical methods [8, 9, 12, 13] have been published in the literature to quantify the magnitude of SL between gene pairs from CDKO screen data. However, most published screens use distinct scoring systems, making comparisons across scoring methods difficult. Currently, there is no consensus on which genetic interaction scoring method yields the best results.

Only a handful of studies have systematically evaluated SL scoring methods, and their scope remains limited. Zamanighomi *et al.* [12], who developed the Gemini genetic interaction scoring method, evaluated multiple scoring methods but they were limited by the lack of a benchmark of true positive and true negative pairs. Moreover, fewer CDKO datasets and scoring systems were available at the time of their publication. Chou *et al.* [14] compared performance of the dLFC (delta log fold change) method and a number of variants on data from four screens using the in4mer library [15] as well as Dede *et al.* [8], Parrish *et al.* [9], and Thompson *et al.* [10] screens. They did not compare against alternative scoring systems such as Gemini [12] or Orthrus [13], which cannot be directly applied to the in4mer screen data. Rather than employing an orthogonal benchmark of SL, as we do here, they evaluated the consistency of SL hits identified across screens using the Jaccard coefficient. The Synthetic Lethality Knowledge Base (SLKB) [16] project consolidates data from multiple CDKO experiments scored using various GI scoring methods. However, SLKB lacks comparative insights into the performance of these methods when evaluated against a ground truth. In contrast, our study evaluates the performance of multiple scoring methods and provides a comparative analysis.

**Figure 1.**
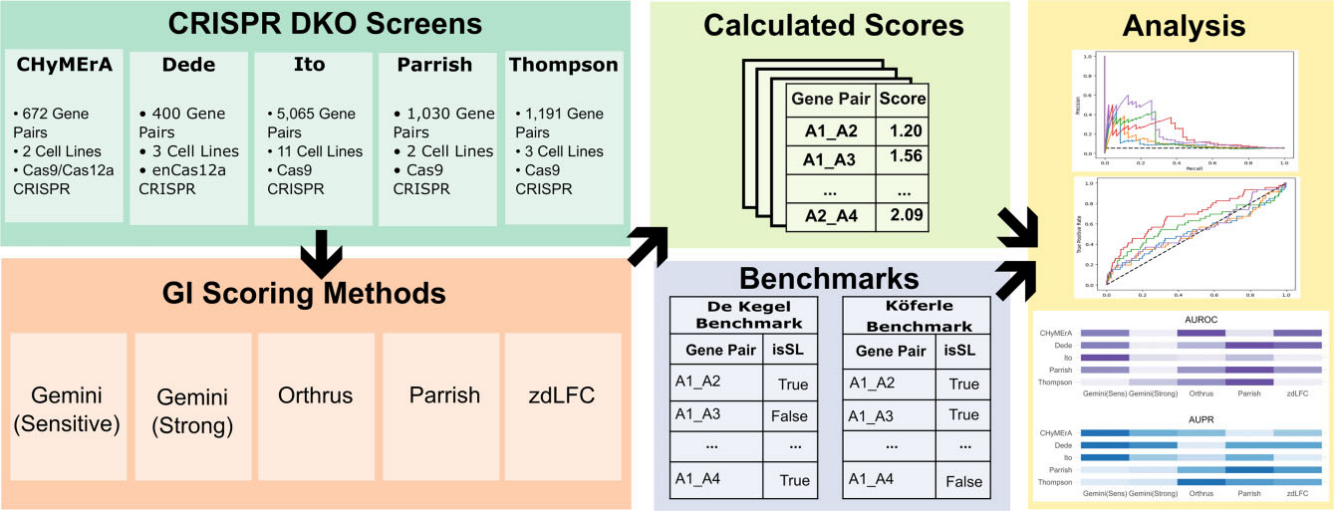
Experiment overview – benchmarking scoring methods for SL detection from CRISPR screen data. Five different CDKO screens are scored for genetic interaction using five different scoring methods. The calculated scores are analysed using two different benchmarks (De Kegel and Köferle benchmarks). AUROC and AUPR for each scoring method on each dataset are calculated and compared.

In this study, we evaluate multiple GI scoring methods using two benchmarks and rate them based on their performance using area under the receiver operating characteristic curve (AUROC) and area under the precision recall curve (AUPR) (Fig. 1). We seek to directly address the question of which GI scoring method performs best. We apply five scoring methods to five CDKO datasets. As benchmarks, we use two different datasets: (i) De Kegel benchmark [17] and (ii) Köferle benchmark [18], detailed in the ‘Benchmark datasets’ section. AUROC and AUPR are computed for each dataset and scoring method combination, and the results are compared across screens.

Overall, our results suggest that the Gemini-Sensitive scoring method consistently ranks higher than other methods across all screens and both benchmark datasets. While the Parrish score also performs reasonably well, Gemini-Sensitive is currently a better first choice due to its availability as an R package with comprehensive user documentation and its ease of application across all screen designs.

## Materials and methods

### CRISPR DKO studies

In pooled CRISPR screens, the fitness associated with a given construct (gRNA or gRNA pair) is typically inferred from the log fold change (LFC) in abundance of that construct between early and late time points, with constructs that cause a growth defect having a negative LFC. Typically, a given gene will be targeted with multiple constructs, and an overall gene effect then inferred using the LFC of all these constructs (referred to as ‘single mutant fitness’ or SMF in this paper). In a combinatorial screen, a pair of genes will be targeted by constructs containing two gRNAs, and the effect of these paired constructs is used to infer ‘double mutant fitness’ or DMF.

We use datasets from five CDKO studies, detailed in Table 1. Each of these screens uses constructs targeting pairs of genes to study DMF and constructs targeting single genes along with a non-targeting control to study SMF. All screens are pooled combinatorial studies. Read counts are measured at both early and later time point(s).

**Table 1.** List of CDKO datasets used in this study, along with their details

Study name	CRISPR approach	Cell lines	# Gene pairs	Early time point (ETP)/control	Late time point (LTP)
Dede	enCas12a	A549, HT29, OVCAR8	400	Plasmid pool	10 population doublings
CHyMErA	Cas9–Cas12a	HAP1, RPE1	672	Day 2	Day 18(HAP1), Day 24 (RPE1)
Ito	Cas9	Meljuso, GI1, MEL202, PK1, MEWO, HS944T, IPC298, A549, HSC5, HS936T, PATU8988S	5065	Plasmid	Day 21
Parrish	Cas9	PC9, HeLa	1030	Immediately post-infection and puromycin selection	12 population doublings
Thompson	Cas9	MEWO, A375, RPE	1191	Day 7 read counts from wild-type A375 cells (Cas9-negative) transduced with the library under similar screening conditions	Day 28

### Genetic interaction scoring methods

A synthetic lethal relationship is inferred when the observed DMF is significantly worse than the expected DMF, which is typically calculated as the product of the SMF of each single-gene knock-out, equivalent to their sum in log space [19]. We use five different GI scoring methods for our analysis: zdLFC, Gemini-Strong, Gemini-Sensitive, Orthrus, and Parrish score (Table 2). These scoring systems primarily differ in how they calculate the expected DMF, and in a variety of other technical details (Table 2).

**Table 2.** List of GI scoring methods analysed in this study, along with their details

**Scoring method**	**Default filtering**	**Default preprocessing**	**Description**	**Implementation**
zdLFC [8]	–	A pseudo-count of 5 is added to all read counts, followed by normalization to an average of 500 reads per guide	Genetic interaction is equal to expected DMF minus the observed DMF. The differences are z-transformed after truncating the top and bottom 2.5% values. zdLFC ≤ −3 indicates SL-Hit	The code is provided as Python notebooks, which can be adapted to different datasets
Gemini-Strong [12]	–	Read counts are normalized to the total number of counts. Normalized read counts are adjusted in a way that that median read count across all guides and all genes is set to 0. A pseudo count of 32 is added	Expected LFC is modelled as a function of sample-dependent as well as sample-independent individual effects of each guide in a pair and the effect of two guides in combination. Uses the coordinate ascent variational inference (CAVI) approach to infer single and double gene effects. The strong variant identifies interactions where the inferred combination effect significantly exceeds either of the individual effects. Captures GIs with ‘high synergy’	Available as an R package with a comprehensive user guide
Gemini-Sensitive [12]	Removes gene pairs of which an individual gene KO results in >50% depletion, compared to gene pairs with the strongest depletion in the screen		The sensitive variant compares the total effect (sum of individual gene effects and the combination effect) with the most lethal individual gene effect. This score can capture ‘modest synergy’	
Orthrus [13]	Removes gRNAs with read count <30 and >10 000. Gene pairs for which there are too few guides remaining post-filtering are also removed	All counts are log_2_-scaled (scaling factor 1e6) and normalized to the total number of read counts. A pseudo count of 1 is added post normalization and scaling	Assumes an additive linear model for the expected LFC of each gene pair in both orientations (A-B/B-A). Estimates effect size by comparing the expected LFC to the observed LFC for each orientation. Can be configured to ignore orientation when needed	Available as an R package with a comprehensive user guide
Parrish score [9]	gRNAs with <2 reads per million in the plasmid pool or a read count of zero at any time point are removed	LFC across guides is scaled such that median of double non-targeting controls is set to 0 and median of positive control pairs (essential genes paired with non-targeting controls) is set to −1	Expected DMF is modelled as the sum of SMF of each gene in the pair. GI score is calculated as the residual of each observed DMF from the control regression line. We refer to it as the Parrish score as we use the implementation provided by Parrish *et al.* [9], but note that this method is a combination of techniques introduced in [20, 21]	The R code was provided by the authors upon request and was adapted for each dataset

These scoring methods are applied on five different CDKO screens: the Thompson screen, the Parrish screen, the Ito screen, the Dede screen, and the CHyMErA screen (Table 1). As each dataset includes distinct sets of gene pairs and cell lines, our approach cannot be used to compare the performance of different screens. Rather, it allows us to assess how well each scoring approach performs on each individual screen.

The Gemini score is the only method that accounts for sample-dependent as well as sample-independent effects and therefore accounts for inherent variability and inter dependencies in combinatorial screens. Sample independent effects account for various sources of variation across screens, such as differences in CRISPR guide activity, promoter strength, and batch effects. However, for studies with multiple cell lines but without common early time point read counts from plasmid pools [7, 9, 22], it is not possible to account for sample independent effects, as each cell line data have to be scored individually.

The key difference between Orthrus and other methods is that Orthrus takes orientation effects into account. This is specifically important for combinatorial CRISPR screens that use two different nucleases to target two genes and may have varying efficacies of guides at each position. An example of such screens is the CHyMErA screen [22], published by the authors of the Orthrus method and discussed in the next section, which uses Cas9 and Cas12a guides to target gene pairs. Even when a single nuclease is used at both positions, guides can have differential effectiveness depending on their position due to variation in promoter efficiency. Orthrus thus accounts for orientation, considering whether a gene is targeted by guide A at position A or by guide B at position B. It performs significance testing on expected and observed sets with matching orientations. However, this requires a reasonable number of guides in each orientation to gain statistical power. Orthrus can be configured to ignore orientation. The difference between enabling or disabling orientation in Orthrus primarily affects the associated *P-*values—for example, a gene pair A-B may be significant, while B-A may not be. However, in our analysis, we use the absolute scores rather than the *P*-values. Therefore, changing this setting would not affect our results.

All studies, except the Parrish study and the Thompson study, target gene pairs in both orientations (A-B and B-A). The Parrish and the Thompson study target gene pairs in a single orientation. This breaks the Orthrus code that needs a gene pair to be in both orientations. To address this, we implemented a workaround: for each gene pair, we duplicated the read counts (e.g. if there were initially 16 rows for each gene pair, there are now 32 rows) and swapped the positions of gene A with gene B, and guide1 with guide2. While this should not affect the final GI score, it may influence the statistical significance, which is not a primary concern in our analysis.

We also observed that the CAVI algorithm used in the Gemini score did not converge for either cell line analysed in the Parrish study. According to the Gemini user guide, the default initialization parameters must be adjusted to achieve convergence in such cases. However, at the time of writing this manuscript, the user guide does not provide further guidance on addressing this issue.

It is also important to note that the implementation of the Parrish score had to be adapted for each study. The original method estimates a single-knock-out (SKO) effect or SMF of a construct (G1_gRNA__ntc1_gRNA_), where G1_gRNA_ is the guide targeting a gene G1 and ntc1_gRNA_ is a non-targeting control guide, using reads from the constructs using the same guide (G1_gRNA_) paired with other non-targeting controls (G1_gRNA__ntc2_gRNA_, …) and mean of the same non-targeting control guide (ntc1_gRNA_) paired with other non-targeting control guides (ntc1_gRNA_ _ ntc2_gRNA_, ntc1_gRNA_ _ ntc3_gRNA, …_). However, in all the studies that we have analysed, except the Parrish study, such additional controls do not exist (e.g. G1_gRNA__ntc2_gRNA_ may be missing) and/or there are no double negative controls (ntc1_gRNA_ _ ntc2_gRNA_, ntc1_gRNA_ _ ntc2_gRNA_, … do not exist). In such cases, we simply set the expected SKO equal to the observed SKO. In CHyMErA and Thompson studies, double negative control do exist; however, the guide sequences do not match those of the single-targeting control guides—only 7.7% of guides in the single-targeting control set overlap with those in the double negative control pairs in the CHyMErA study and none overlap in the Thompson study. Hence, for every study except the Parrish study, the observed value of SKO was used to estimate the expected value of double KO (G1_gRNA__ G2_gRNA_) using the linear modelling approach outlined in [9].

It is also important to note that most methods outlined above use distinct filters for guides based on read counts or single gene essentiality. For our primary analysis, we omitted all such default steps to ensure consistent preprocessing across all methods and datasets. It is also worth noting that only Gemini and Orthrus are available in the form of R packages, with comprehensive user guide documentation. This makes them easier to run compared to other methods. For all other methods, the code provided by the authors had to be adapted for each dataset.

### Benchmark datasets

To evaluate each scoring method on the CDKO datasets, a benchmark set of pairs labelled as SL or non-SL is necessary. Since no established gold-standard exists for SL, we use two compiled lists of SL interactions derived from single-gene knock-out CRISPR screens as benchmarks: De Kegel benchmark [17] and Köferle benchmark [18]. Both lists are derived from analysis of single-gene CRISPR screens across a panel of hundreds of cell lines and therefore should be robust across various cancer types and genetic backgrounds.

**Figure 2.**
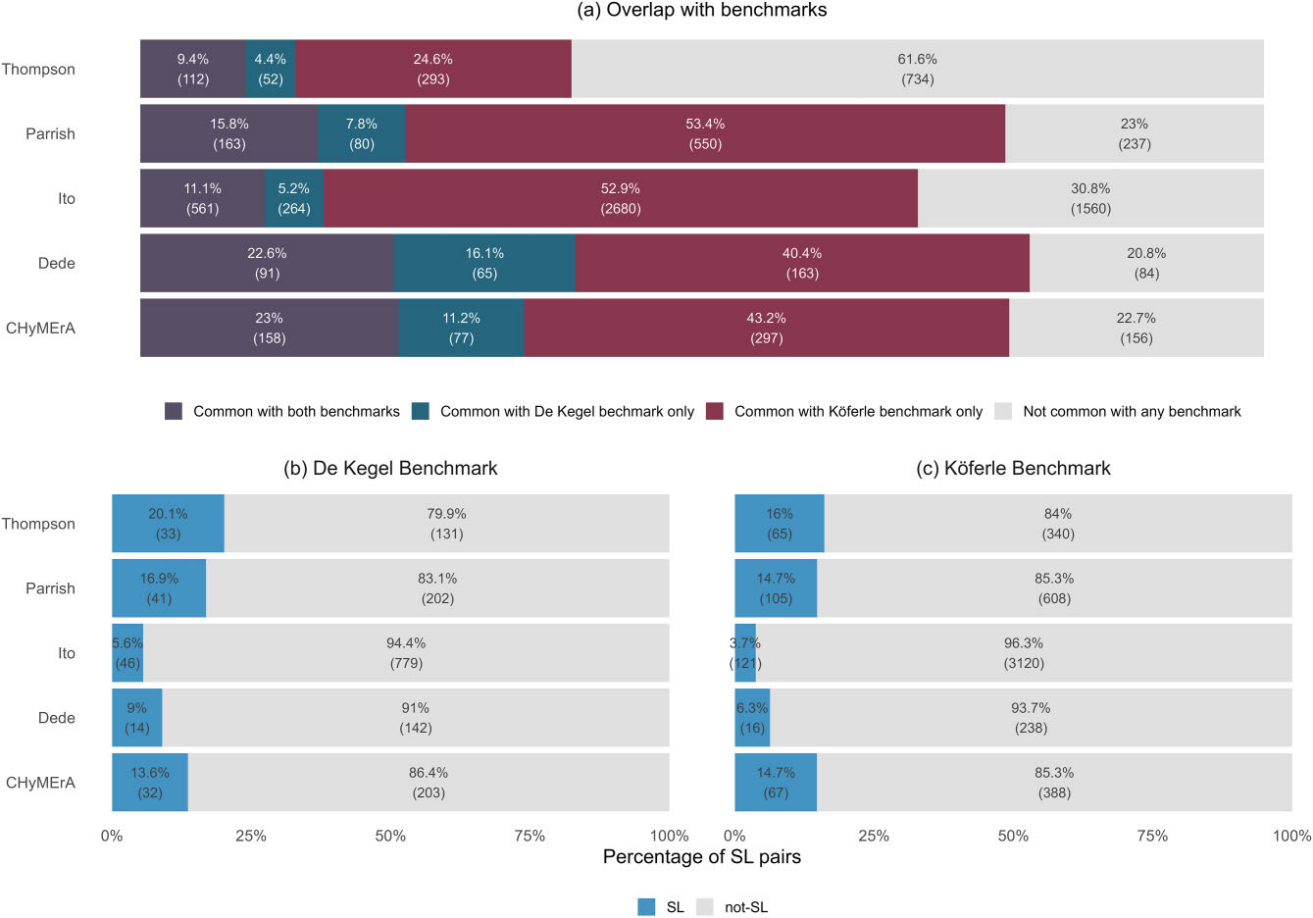
Analysis of the gene pairs in CRISPR DKO studies and benchmark datasets. (**A**) Proportion of gene pairs in each study common with both benchmarks, only De Kegel benchmark and Köferle benchmark. Percentage of gene pairs of each study that are identified as SL hits in (**B**) the De Kegel benchmark and (**C**) Köferle benchmark.

*De Kegel benchma* *rk*. This list contains SL annotations for 3,634 paralog pairs, with roughly 3.5% labelled as SL and the remainder non-SL. SL calls were made in [17] by analysing CRISPR screens in 768 cancer cell lines—a linear model was used to assess the association between loss of one gene and sensitivity to the inhibition of its paralog. Loss of a gene was based on three criteria: homozygous deletion, loss-of-function mutation, or loss of mRNA expression. Due to a variety of restrictions on the paralog pairs tested (e.g. minimum sequence identity threshold, broad expression, and gene loss in at least 10 cell lines) this dataset only annotates around 10% of the 36.6k protein coding paralog pairs from Ensembl used in [17]. Each study in our analysis has a different proportion of overlapping gene pairs with this dataset as shown in Fig. 2A.

*Köferle benchmark*. This list contains annotations for 48,095 pairs, with 0.92% labelled as SL and the remainder non-SL. This list was generated using the same resources as the De Kegel benchmark [17]; however, the associations were calculated by correlating the CRISPR dependency scores of one paralog and the mRNA expression of another. A ‘hit’ was identified when the Spearman’s correlation coefficient for a gene pair deviates by more than three standard deviations from the mean of all gene pairs and its corresponding *P*-value was <0.05. For our purposes, we define SL pairs as the subset of hits where the Spearman correlation is >0 (indicating decreased expression of one results in increased dependency of the other). Non-SL pairs are those where the Spearman’s correlation coefficient is <0 and the pair is not labelled as a hit. For our study, we filter the pairs where the depletion/dependency score is taken from DepMap ‘AVANA’ screens [23], as this dataset contains the largest proportion (46%) of the candidate biomarkers and is the most comprehensive in terms of the number of cell lines included. Compared to the De Kegel benchmark, this dataset has a higher percentage of overlapping gene pairs for each study as shown in Fig. 2A.

Figure 2B and C show the percentage of positive SL pairs out of total for both benchmark datasets. It is evident that all datasets are highly imbalanced, particularly the Ito study, where the share of positive SL pairs is significantly lower compared to others.

A total of 2,347 gene pairs are common to both the De Kegel and Köferle benchmarks. Among these, 2,222 pairs are consistently labelled as non-SL, while 61 pairs are consistently labelled as SL in both benchmarks. Discrepancies occur for 22 pairs, which are labelled as SL only in the De Kegel benchmark, and 42 pairs, which are labelled as SL only in the Köferle benchmark. A Fisher’s exact test on the resulting contingency table yields a highly significant *P*-value (<2.2 × 10^−16^), with an odds ratio of 144.72, indicating a strong concordance between the two benchmarks in SL classification.

### Evaluation metrics

To evaluate and compare the performance of each scoring method, we report two threshold-free measures: AUROC and AUPR. These metrics are computed using the trapezoidal rule with the Python scikit-learn package (version 1.5.2).

An ROC curve shows the trade-off between the false positive rate and true positive rate of a binary classifier [24]. AUROC of 0.5 indicates a random classifier. For imbalanced datasets, like we have, the PR curve is often more informative than the ROC [25] as it shows a trade-off between precision and recall across varying threshold settings. The baseline performance in a PR curve is represented by a horizontal line equal to the ratio of positive samples to total samples.

As noted earlier, each study screens a distinct subset of gene pairs, and there is significant variation in class imbalance across studies. For example, only 5.6% of gene pairs screened in the Ito study and present in the De Kegel benchmark are annotated as SL, whereas in the Thompson study, 20.1% of the overlapping pairs are annotated as SL (Fig. 2B). Therefore, the expected precision of a random classifier is inherently higher in the Thompson study than in the Ito study. This highlights that performance comparisons are meaningful within studies, but not across studies.

For each dataset, the method with highest AUPR and highest AUROC is reported with both benchmarks. To assess the robustness and statistical significance of the highest performing method, we conducted a subsampling-based evaluation. We performed stratified sampling by randomly selecting 75% of the dataset without replacement, while preserving the original class distribution of the ground truth labels. For each subsample, we computed the AUROC and AUPR for each method. This procedure was repeated 500 times, yielding a distribution of performance scores for each method. To test whether the method with the highest mean AUPR/mean AUROC method was significantly better than the others, we performed paired *t*-tests comparing its performance to that of each competing method across the 500 replicates. *P*-values were corrected for multiple comparisons using the Bonferroni correction, and a method was considered significantly different if the adjusted *P*-value was <0.05.

### Default filtering settings of GI scoring methods

As noted in Table 2, each method returns a different number of scored pairs due to varying default filtering settings. To ensure a fair comparison, we standardized the methods by removing these preprocessing steps, forcing them to score all pairs regardless of low read counts or whether one of the genes might be essential. The Gemini-Sensitive essentiality filter removed 10%–20% of gene pairs in all screens except the Dede screen and Orthrus default filter removed almost 50% of the pairs in the CHyMErA screen (Supplementary Fig. S1). Therefore, we also include an analysis of how the filtering steps of the Gemini-Sensitive score and the Orthrus score impact the overall results.

## Results

### All scoring methods are correlated

**Figure 3.**
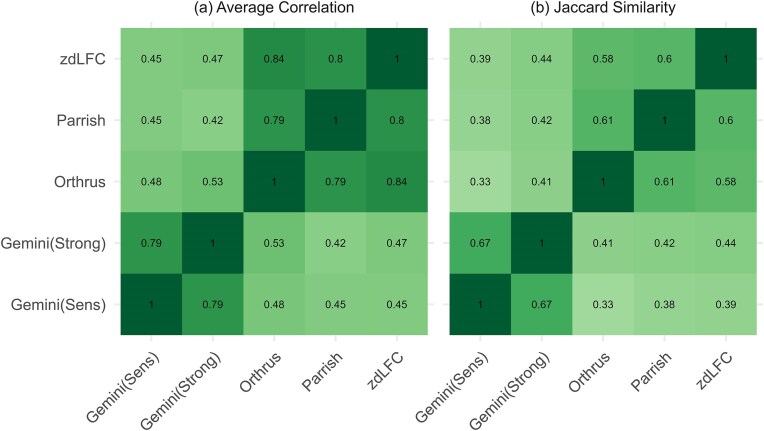
Agreement between scores calculated by different methods. (**A**) Average correlation across methods for all studies. (**B**) Average Jaccard similarity of top 5% scored gene pairs across methods. Darkest shade of green shows an average correlation of 1 or average Jaccard index of 1.

We first applied each scoring system to the results of each screen in each dataset, resulting in a total of 105 scored datasets encompassing 21 individual cell line screens. This allowed us to assess the agreement between scores generated by different approaches, presented in Fig. 3, aggregated over all screens, with the comparisons across individual screens presented in Supplementary Fig. S2. As might be expected, the scores generated by all scoring approaches are at least moderately correlated (minimum Spearman correlation of 0.42) (Fig. 3A). Two clear groupings emerge from this analysis—one group containing the two Gemini variants (sensitive and strong) and a second group containing the three other scores (Orthrus, Parrish, and zdLFC). Gemini is conceptually different from the other three scores in that it uses a Bayesian approach to estimate single and combination effects, while accounting for guide specific variation in efficiency and cell line specific effects. GIs are then scored using these inferred combination and single gene effects, with the difference between the strong and sensitive scores being in how they are subsequently processed. In contrast, the other three methods primarily model the expected DMF as a linear function of the observed SMF and compare this expected value with the observed DMF.

As only a minority of gene pairs are expected to display GIs, and hence most scores may be close to zero, we also analysed the agreement between the top 5% of gene pairs identified by each scoring system (i.e. the pairs with the most negative genetic interaction effect, indicative of SL). To quantify the agreement between scoring systems, we used the Jaccard index—the size of the intersection between the hits identified by both systems divided by the size of the union of the hits, with a value of 0 indicating no overlap and 1 indicating complete overlap (Fig. 3B). Consistent with our correlation analysis, the scoring systems fell into two distinct groups with Gemini-Sensitive and Gemini-Strong scores in one group with high Jaccard scores between them and Orthrus, zdLFC, and Parrish in the other. While there is variation in the average Jaccard across different studies (Supplementary Fig. S2B) these groupings are generally consistent.

### The Gemini-Sensitive score yields higher AUPR compared to others using De Kegel benchmark

We next analysed the performance of each scoring system across each study using the benchmark datasets. For our analysis, we computed AUROC and AUPR metrics by combining all cell lines within each study, and also by analysing each cell line individually within each study. We focus primarily on performance aggregated by study rather than by cell line, to avoid a single study [11] with a large number of cell lines dominating the results. We then assessed the performance of each score–study combination, as well as each score-cell line combination using the two benchmarks.

**Figure 4.**
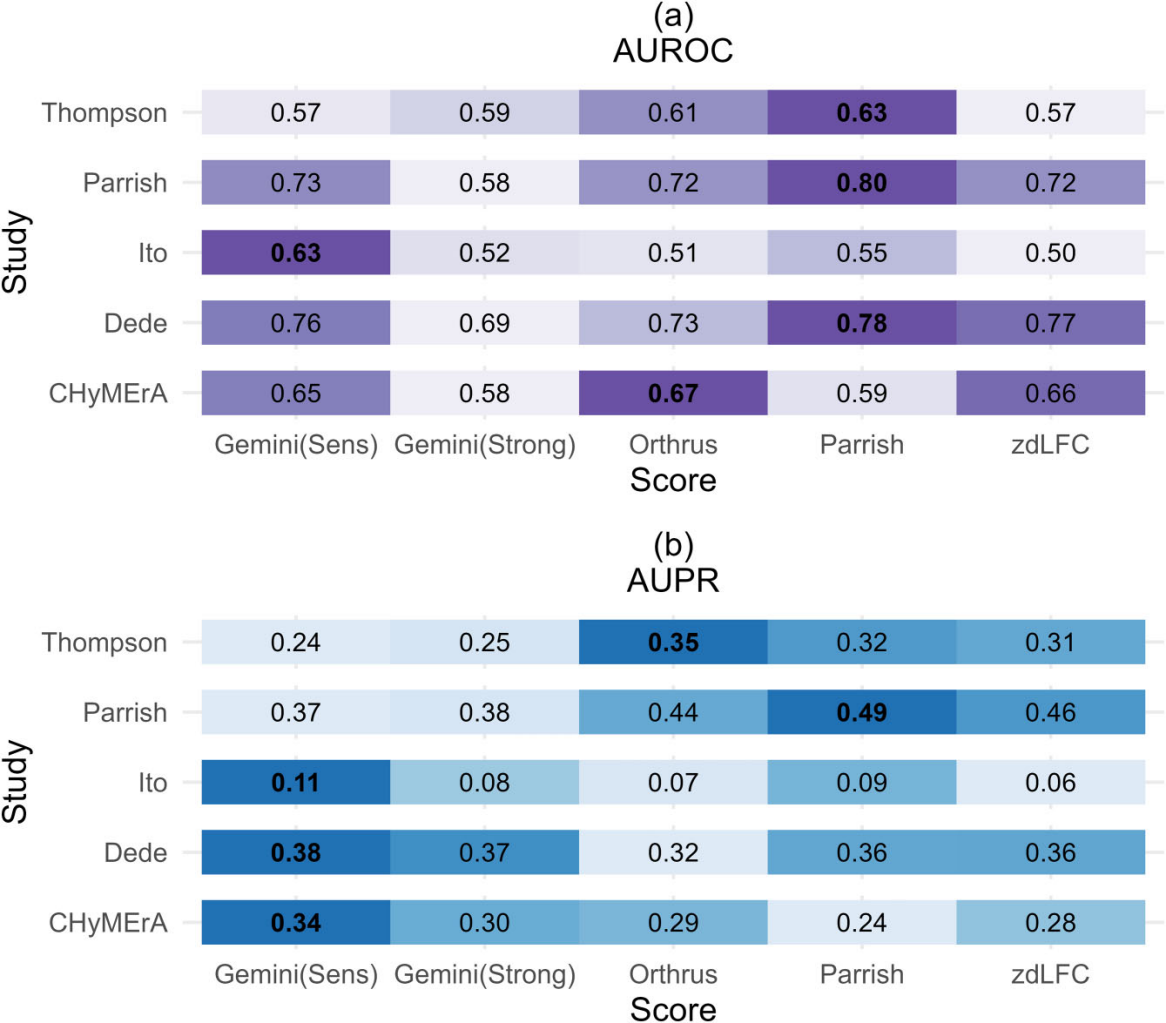
Evaluation of scoring systems using the De Kegel benchmark. (**A**) AUPR and (**B**) AUROC of each study with all cell lines combined. The colour gradients are study specific. The colour represents the normalized AUROC/AUPR for each study, with the darkest shade of purple/blue indicating the highest AUROC/AUPR within that study. As each study has different baseline precision rates, this graph should be used to compare scoring methods across an individual study rather than across studies.

Evident from Fig. 4A, the Parrish score achieves the highest AUROC in three of the studies: the Thompson study, the Parrish study and the Dede study. Gemini-Sensitive score yields the highest AUROC for the Ito study and the Orthrus score yields the highest AUROC for the CHyMErA study. We note that the Gemini score was developed by authors of the Ito study, Orthrus was developed by the authors of the CHyMErA study, zdLFC was developed by the authors of the Dede study, while Parrish *et al.* [9] adapted previous scoring systems to apply to their own screens. In this case, therefore, the highest AUROC generally is observed when the method developed by a group of authors is paired with the screen performed by those authors.

As shown in Fig. 4B, the Parrish score achieves the highest AUPR in one out of five datasets (Parrish), whereas Gemini-Sensitive leads in three (Ito, Dede and CHyMErA). For each study, we found that the top-scoring method was significantly better than all other methods (adjusted *P*-value < .05). It is worth noting that, due to class imbalance (Fig. 2B and C), the expected AUPR for a random classifier is low in each case (e.g. 0.056 for the Ito study).

When analysing individual cell lines, the Gemini-Sensitive score yields the highest AUROC for 12 out of 21 cell lines (primarily those from the Ito study), followed by the Parrish score that yields the highest AUROC for 6 out of 21 cell lines (primarily those from Parrish, Dede, and Thompson) (Supplementary Fig. S3). The Gemini-Sensitive score yields highest AUPR for 11 cell lines (primarily Ito), the Parrish score yields highest AUPR for 5 cell lines (including both screened by Parrish), and the Orthrus score yields the highest AUPR for 4 cell lines (Supplementary Fig. S3).

### The Gemini-Sensitive score consistently yields higher AUPR and AUROC compared to others using Köferle benchmark

**Figure 5.**
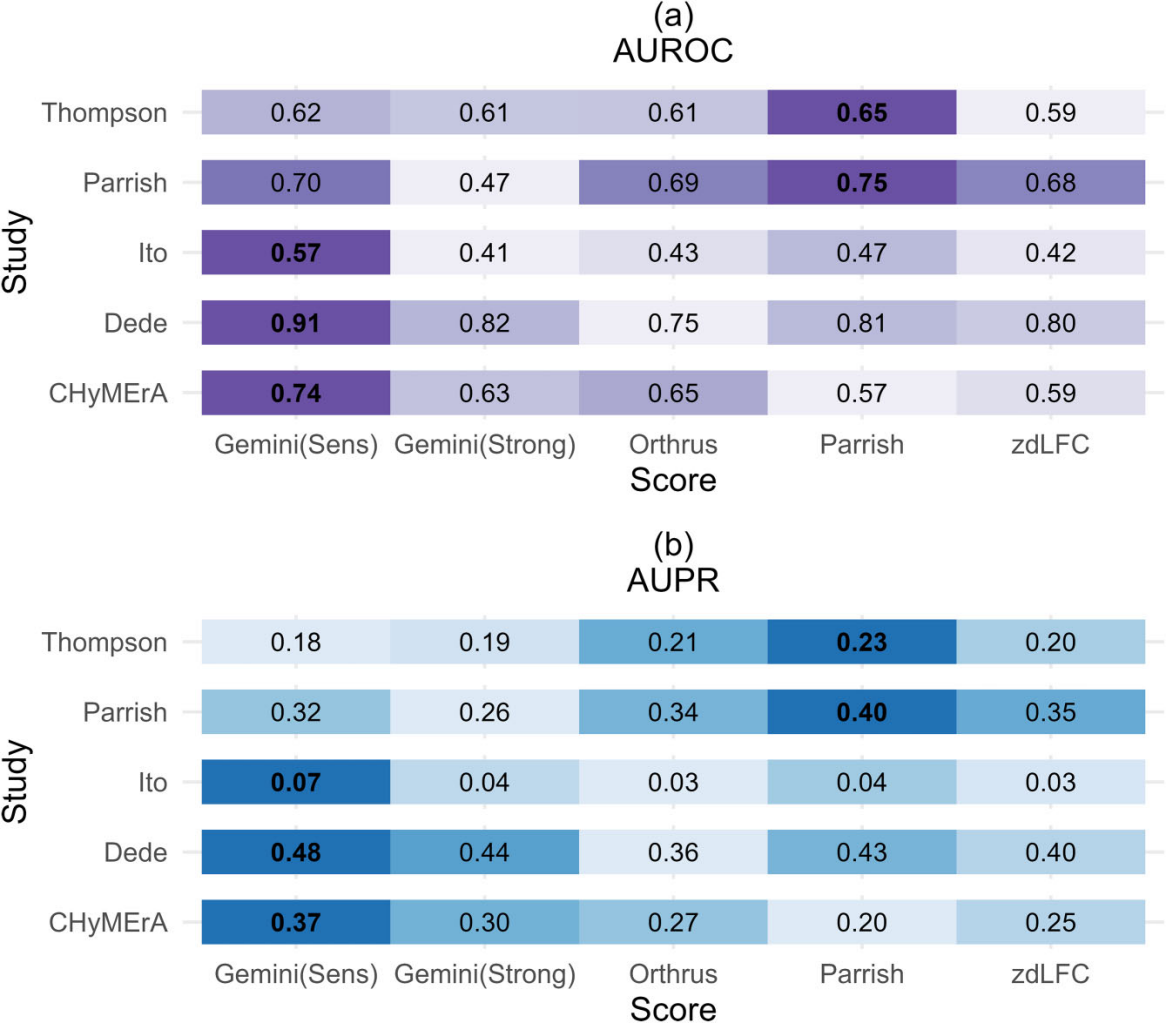
Evaluation of scoring systems using the Köferle benchmark. (**A**) AUPR and (**B**) AUROC of each study with all cell lines combined. The colour gradients are study specific. The colour represents the normalized AUROC/AUPR for each study, with the darkest shade of purple/blue indicating the highest AUROC/AUPR within the corresponding study. As each study has different baseline precision rates, this graph should be used to compare scoring methods across an individual study rather than across studies.

Figure 5A shows the Gemini-Sensitive scores achieving the highest AUROC and AUPR in three studies (Ito, Dede, and CHyMErA), the Parrish score achieves the highest AUROC and AUPR in two studies (Thompson and Parrish) (Fig. 5B). As mentioned before, due to class imbalance (Fig. 2B and C), the baseline AUPR is low in each case. These results are mirrored when analysing individual cell lines, where the Gemini-Sensitive score has generally the highest AUPR and AUROC scores in cell lines from the Ito, Dede, and CHyMErA screens, and the Parrish score has the highest AUPR and AUROC scores for those from Parrish and Thompson screens (Supplementary Fig. S4). As in the previous section, statistically significant differences (adjusted *P* < .05) confirmed that the top method outperformed all others in each study.

The Parrish score performs best on the Parrish screen and the Thompson screen using both benchmarks with respect to AUROC (Figs 4 and 5). In case of AUPR, the Parrish score performs best for the Parrish screen when the De Kegel benchmark is used and performs best for both the Thompson screen and the Parrish screen with the Köferle benchmark. Both studies have an asymmetric guide layout, meaning that the combination of genes A and B is knocked out in only one orientation (A-B), with no (B-A) orientation, suggesting that the Parrish score may be the best scoring system in this setting.

### The default essential gene filter in Gemini-Sensitive score improves results

**Figure 6.**
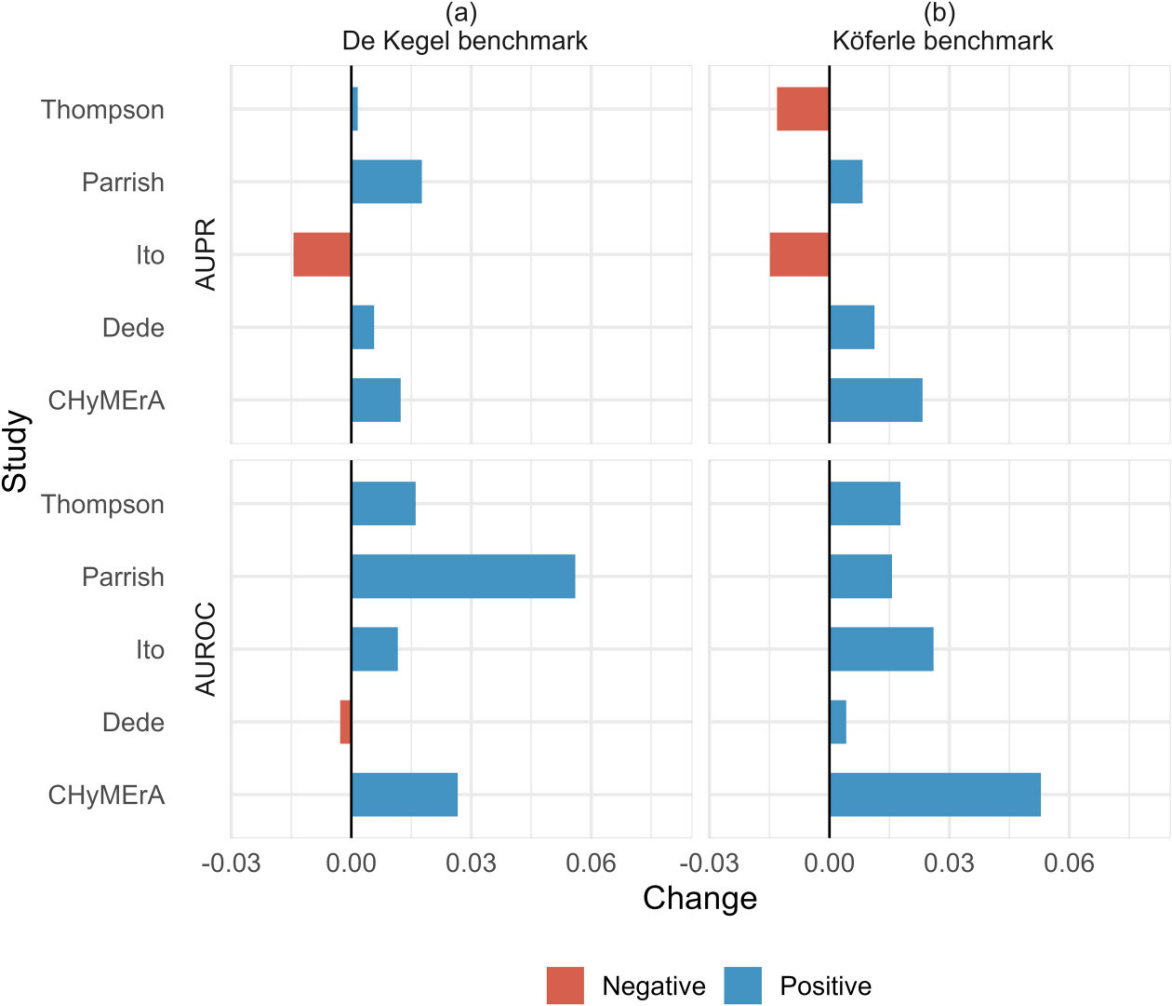
Gemini filtering step generally improves performance. Panels (**A**) and (**B**) show the changes in AUPR and AUROC for each study when the essential gene filter of Gemini-Sensitive scoring method is applied versus not applied, using (A) De Kegel and (B) Köferle benchmarks. A positive value indicates that the filtering step improves the results.

The default filtering step in the Gemini-Sensitive score drops a significant number of gene pairs (between 0.4% and 20%) across the studies (Supplementary Fig. S1). We assessed the impact of this filtering on AUROC and AUPR for each study. AUROC and AUPR values of the filtered datasets are generally higher compared to the unfiltered datasets (Fig. 6A and B), when evaluated against both benchmarks. This indicates that the single gene essentiality filter helps to reduce false positives and hence improves AUPR and AUROC. However, the overall improvement is modest, with a maximum increase of 5% for AUROC and 3% for AUPR.

We performed an additional analysis to see if there is a link between the number of essential genes dropped by the Gemini-Sensitive score and the improvement in its performance when default filtering step is applied (Supplementary Fig. S5). We observe a mild correlation here, suggesting that screens with a larger number of essential genes may benefit from Gemini’s default filtering step.

### The default read count filter of Orthrus discards usable information

**Figure 7.**
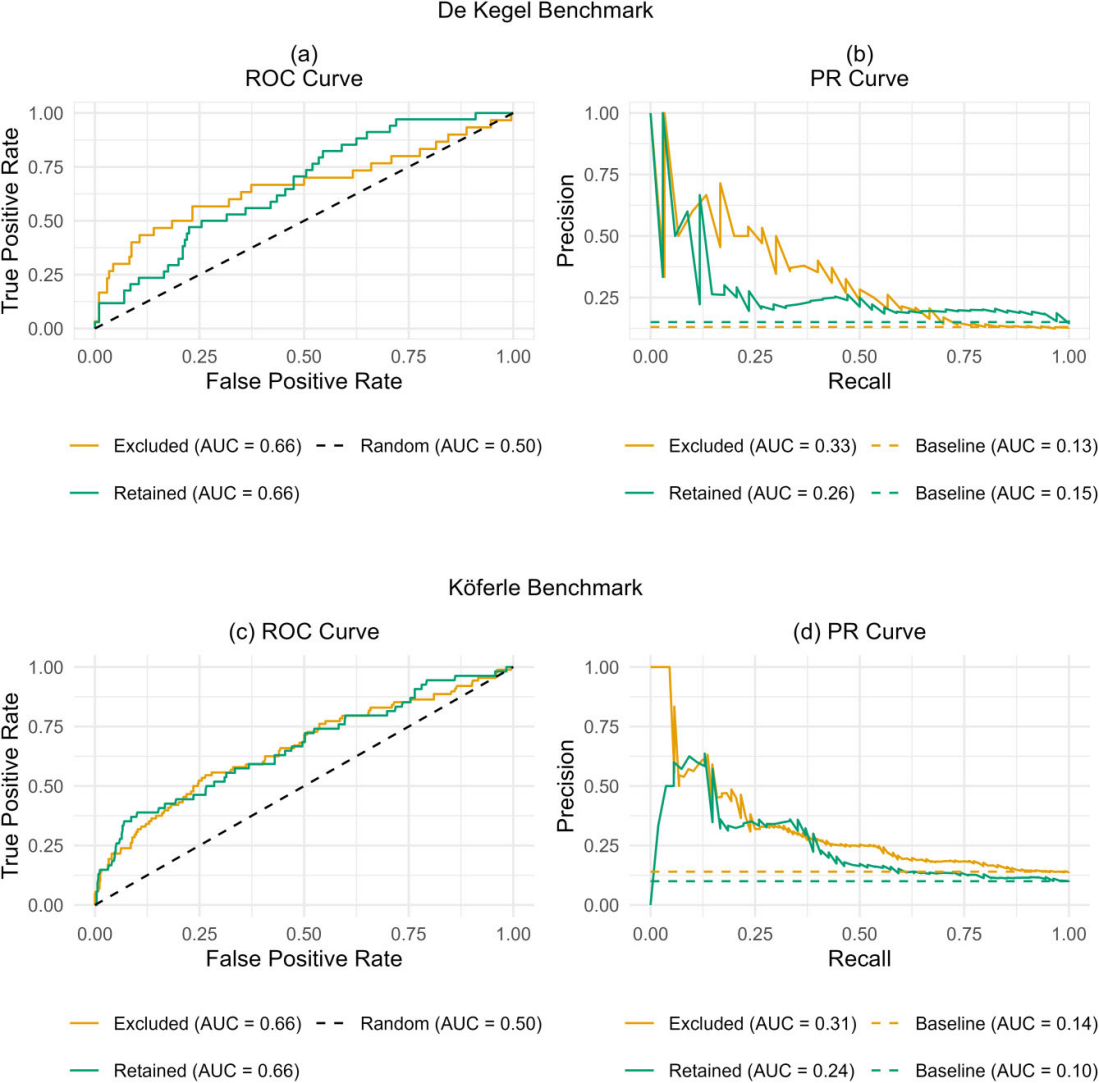
Impact of Orthrus default filtering steps. (**A**) ROC curve and (**B**) PR curve of Orthrus score on CHyMErA pairs that are excluded (*n*= 236), after default filtering step versus CHyMErA pairs that are retained (*n* = 234) using the De Kegel benchmark. (**C**) ROC curve and (**D**) PR Curve of Orthrus score on CHyMErA pairs that are excluded (*n* = 636), after default filtering step versus CHyMErA pairs that are retained (*n* = 538) out using the Köferle benchmark.

The Orthrus default filtering steps result in almost 50% genes from the CHyMErA dataset being excluded from analysis (Supplementary Fig. S1). We compared the AUROC and AUPR of gene pairs that were excluded after the Orthrus default filter was applied, (*n* = 236) with those that were retained (*n* = 234) (Fig. 7A and B). Interestingly, while the AUROC of both sets was similar, the AUPR of the excluded gene pairs was significantly higher than that of the retained pairs. This suggests that removing gene pairs based on a small number of guides remaining post-filtering may discard valuable information, as these filtered-out pairs still perform well on both benchmarks. Similar observations were made when Köferle benchmark was used (Fig. 7C and D).

## Discussion

Identifying SL is invaluable in cancer therapy research; however, determining the most suitable statistical method to accurately identify SL from a CRISPR screen remains highly challenging.

Here, we have performed the first systematic evaluation of the existing GI scoring methods for CDKO screens. We have used five different scoring methods on five different CRISPR DKO studies and evaluated the performance of each scoring method on each study using two benchmarks. We found that the results from all methods exhibit moderate to strong correlation. In particular, Gemini-Sensitive is highly correlated with Gemini-Strong, and both Gemini variants have comparatively moderate correlation with rest of the methods. The other three methods—zdLFC, Parrish, and Orthrus—model the expected LFC as a linear combination of observed individual gene knock-out effects. In contrast, Gemini employs a variational Bayesian approach to model the observed LFC as a function of both sample-independent and sample-dependent individual effects of each guide in a pair and the effect of two guides in combination. The Gemini-Strong variant compares the combination effect of a gene pair to the most pronounced individual effect (flags an interaction when the combination effect exceeds the effect of either gene), while Gemini-Sensitive compares the total effect (the sum of the individual gene effects and the combination effect) to the most lethal individual gene effect.

In most scenarios, either the Gemini-Sensitive score or the Parrish score yields the highest AUROC and AUPR (Figs 4 and 5). Given that AUPR is the more appropriate metric for imbalanced datasets such as ours, we conclude that the GEMINI-Sensitive score outperforms all other methods in the majority of cases (three out of five studies). The Parrish score gives superior results on datasets that use an asymmetric guide orientation and should therefore be used on such datasets. However, if we consider user-friendliness, installation procedures, code availability, reproducibility, and documentation quality as secondary measures [26], Gemini-Sensitive is more user-friendly compared to the Parrish score as it comes as an easy-to-use R package with comprehensive documentation. Our implementation of the Parrish score was based on code provided by the authors and we had to make a number of adaptations to the code provided by Parrish *et al.* [9] to make it work with screens lacking double negative controls. While this paper was under review, an R package implementing the Parrish score, called ‘gimap’ [27], was made available by the authors. The current version of this package does not support screens lacking double negative controls, which prevents application to the any of the studies we analyse except the Parrish study. As a result, we have not benchmarked this implementation. However, future updates to the package may enable compatibility with such screens.

One current drawback of using the Gemini score is that no instructions are available to adjust the model initialisation parameters for the models that do not converge with default parameters.

We observe a slight hint of home ground advantage in Fig. 4, where the highest AUROC is generally achieved when a method is applied to the screen conducted by the same group that developed it (Orthrus scores highest for CHyMErA, Parrish scores highest for the Parrish study and Gemini-Sensitive scores highest for the Ito study). However, this is not always the case—for instance, Fig. 5 shows that Orthrus does not perform best on the CHyMErA screen, and zdLFC does not perform best on the Dede screen when the Köferle benchmark is used.

We also conduct a secondary analysis on the impact of default filtering steps of the Gemini-Sensitive and the Orthrus methods. We found that in the case of Gemini-Sensitive score, filtering essential gene pairs as recommended by the developers prior to scoring results in an increase of AUPR and AUROC in most studies (Fig. 6). We also found that the default filtering step of the Orthrus scoring system discards usable information; i.e. AUPR of unfiltered scored pairs is higher compared to AUPR of filtered scored pairs (Fig. 7).

Although both benchmarks are derived from largely the same input data, there are some conceptual differences between the approaches that result in different synthetic lethal calls and a significantly different search space. Köferle *et al.* [18] systematically assessed the correlation between the expression of one gene (biomarker) and the CRISPR score of another (target) for all paralog pairs with available expression and CRISPR data. A pair can be identified as synthetic lethal where there is a significant association between the expression of the biomarker and the CRISPR score of the target, even if the target score does not indicate a significant growth defect. In contrast, De Kegel *et al.* [17] restricted their analysis to recurrently lost biomarker genes (silenced/mutated/deleted in at least 10 cell lines), pairs of broadly expressed genes, and only identify synthetic lethal for pairs where a significant fitness defect is observed for the target in at least one cell line. Consequently, there is some discordance between the synthetic lethal calls and a larger difference between the pairs analysed by the two approaches.

Obviously, the results we report are dependent on the benchmarks used, and as additional benchmarks become available it will be possible to extend our analyses. While this manuscript was in review, a new dataset (termed BaCoN) was published [28], which is conceptually similar to the Köferle *et al.* [18] analysis and uses the same underlying datasets but corrects for some systematic issues using an improved computational approach. We treated the 808 high-confidence gene pairs reported by BaCoN as SL and considered all other pairs as non-SL. Regardless of whether Gene A buffers Gene B or vice versa, we consider the pair to be synthetic lethal if buffering exists in at least one direction. This analysis yielded results consistent with our original findings (Supplementary Figs S6 and S7). A large-scale genetic interaction map [29], generated by screening a genome-wide CRISPR library in isogenic knock-out cell lines rather than combinatorial CRISPR screening, may provide additional alternative benchmarks.

In general, GIs can be both negative (double perturbation has worse growth than expected) and positive (double perturbation has better growth than expected) [19]. Although both types of interactions can be revealed by combinatorial CRISPR screens, our analysis here has solely focussed on the evaluation of extreme negative GIs, i.e. synthetic lethal pairs. Currently, most combinatorial screens are performed to identify new synthetic lethal targets and so our approach allows us to evaluate the most common use case. Furthermore, the Köferle and De Kegel datasets provide reasonable benchmarks. However, positive GIs can reveal interesting biological relationships between genes [9], and therefore, benchmarking scoring systems for the ability to identify such pairs would be useful. An open challenge is the identification of a suitable benchmark—in model organisms, it has been well established that positive interactions are enriched among members of the same protein complex [30] but it is unclear whether this is the most suitable benchmark for tumour cell lines where positive GIs have primarily been reported between pairs where at least one gene is a tumour suppressor [9].

Here, we have evaluated combinatorial CRISPR screens that assess SL between carefully selected gene pairs—paralog pairs. In this scenario, each gene may only be assessed for genetic interaction with one other gene (its paralog) and hence the estimation of single gene effects is heavily dependent on control constructs. In contrast to this approach, others have made use of matrix screens, where sets of up to hundreds of genes are screened in an all-against-all fashion, such as Horlbeck *et al.* [31]. Because every individual gene in these studies is tested in hundreds of combinations, it may be possible to gain an improved estimate of the size and variance of single gene effects from all double perturbations rather than relying on estimates from a small number of control constructs, as has been done in yeast [32]. Therefore, it may also be worth evaluating different scoring systems in this setting, but the challenge will be the identification of a suitable benchmark.

## Supplementary Material

lqaf129_Supplemental_File

## Data Availability

All analysis and code for reproducing the results of the study are publicly available at https://figshare.com/s/f3bff98db72e1039414b and https://github.com/cancergenetics/Benchmarking-GI-Scores. The results of applying each scoring method to each screen are available at https://figshare.com/s/59ee190b1879fe3eb191.
