# Supplementary material for: Benchmarking genetic interaction scoring methods for identifying synthetic lethality from combinatorial CRISPR screens
Source: NAR Genom Bioinform. 2025 Sep 26;7(3):lqaf129. doi: 10.1093/nargab/lqaf129 (PMC12464814; doi:10.1093/nargab/lqaf129)
Supplement: lqaf129_Supplemental_File [file lqaf129_supplemental_file.docx]

**SUPPLEMENTARY FIGURES**

**Benchmarking Genetic Interaction Scoring Methods for Identifying Synthetic Lethality from Combinatorial CRISPR Screens**

**AUTHORS**

Hamda Ajmal^1,2,4^, Sutanu Nandi^1,2,3,4^, Narod Kebabci^1,2,5^, Colm J. Ryan^1,2,3,4*^

^1^ Conway Institute of Biomolecular and Biomedical Research, University College Dublin, Dublin, D04 V1W8, Ireland

^2^ School of Computer Science, University College Dublin, Dublin, D04 V1W8, Ireland

^3^ Systems Biology Ireland, University College Dublin, Dublin, D04 V1W8, Ireland

^4^ School of Medicine, University College Dublin, Dublin, D04 V1W8, Ireland

^5^ The Research Ireland Centre for Research Training in Genomics Data Science, University College Dublin, Dublin, D04 V1W8, Ireland

* To whom correspondence should be addressed. Email: [colm.ryan@ucd.ie](mailto:colm.ryan@ucd.ie).

**
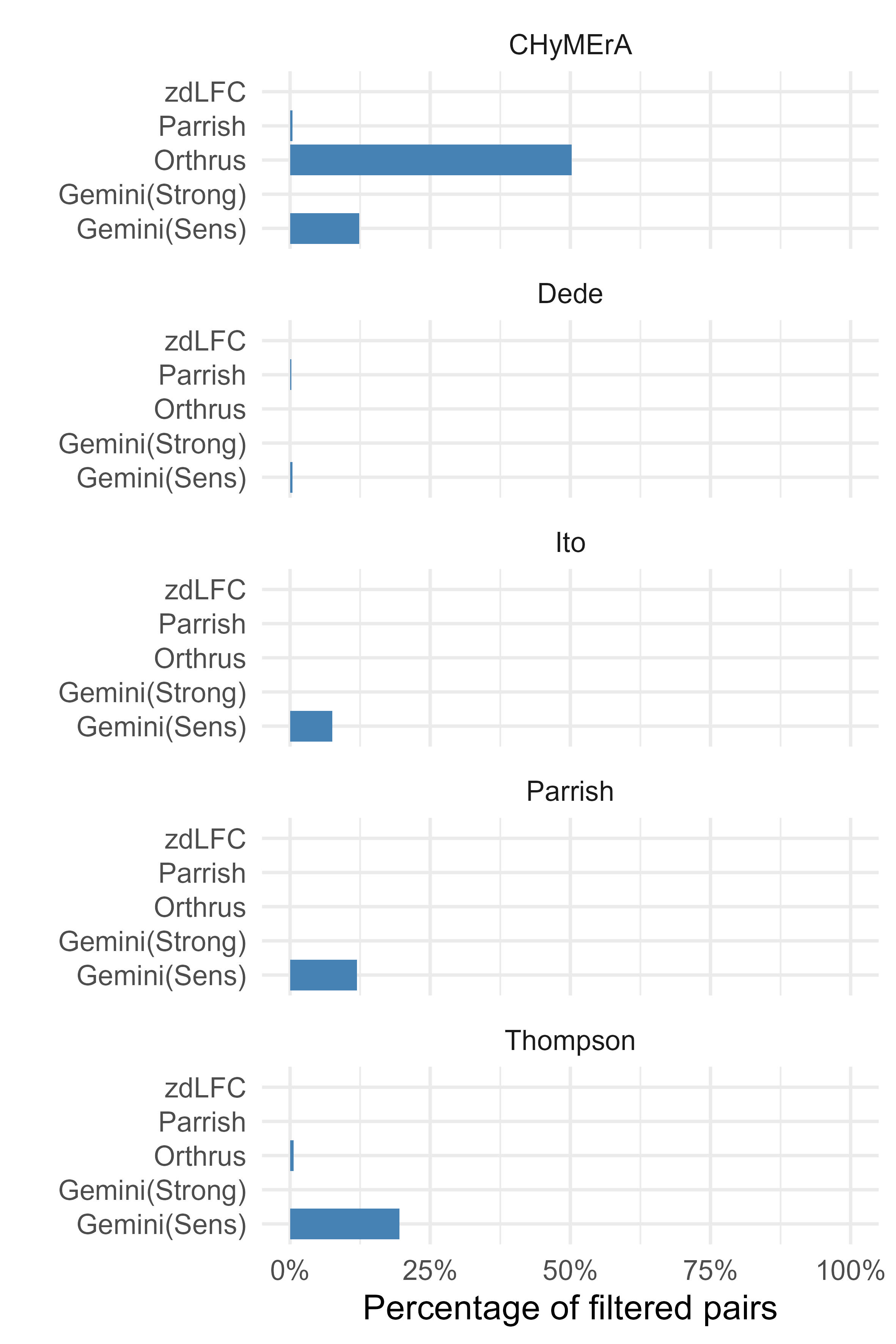
**

**Figure S1**. Percentage of gene pairs filtered by each method when default filters are applied.





**Figure S2.** Comparison of Scoring Methods (a) Average correlation of scoring methods within each study; (b) Average Jaccard similarity of the top 5% scored gene pairs across scoring methods within each study.


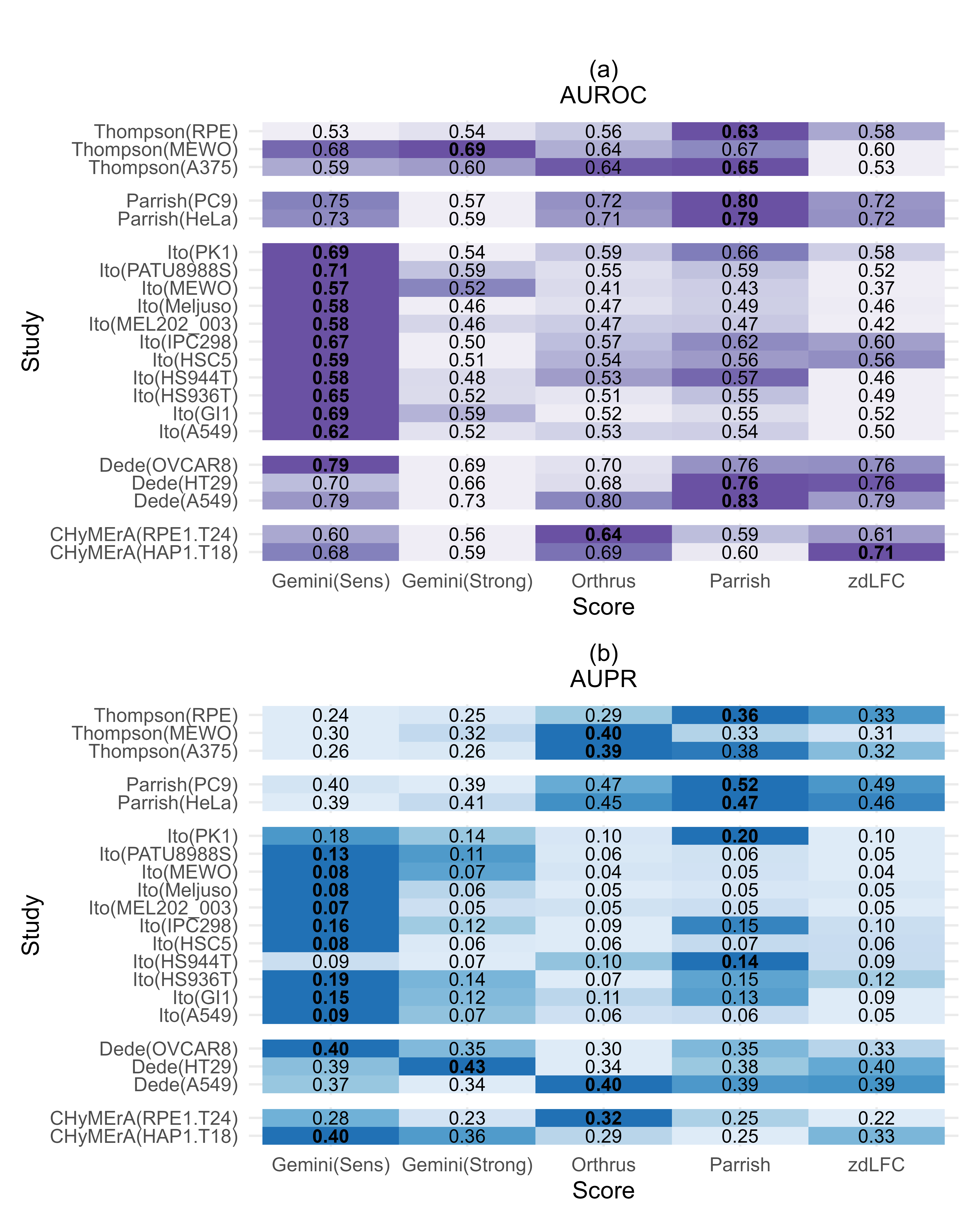


**Figure S3.** Evaluating scoring systems using the De Kegel benchmark. (a) AUROC and (b) AUPR across individual cell lines of each study using De Kegel benchmark. The colour gradients are study specific. As each study has different baseline precision rates, this graph should be used to compare scoring methods across an individual study rather than across studies.


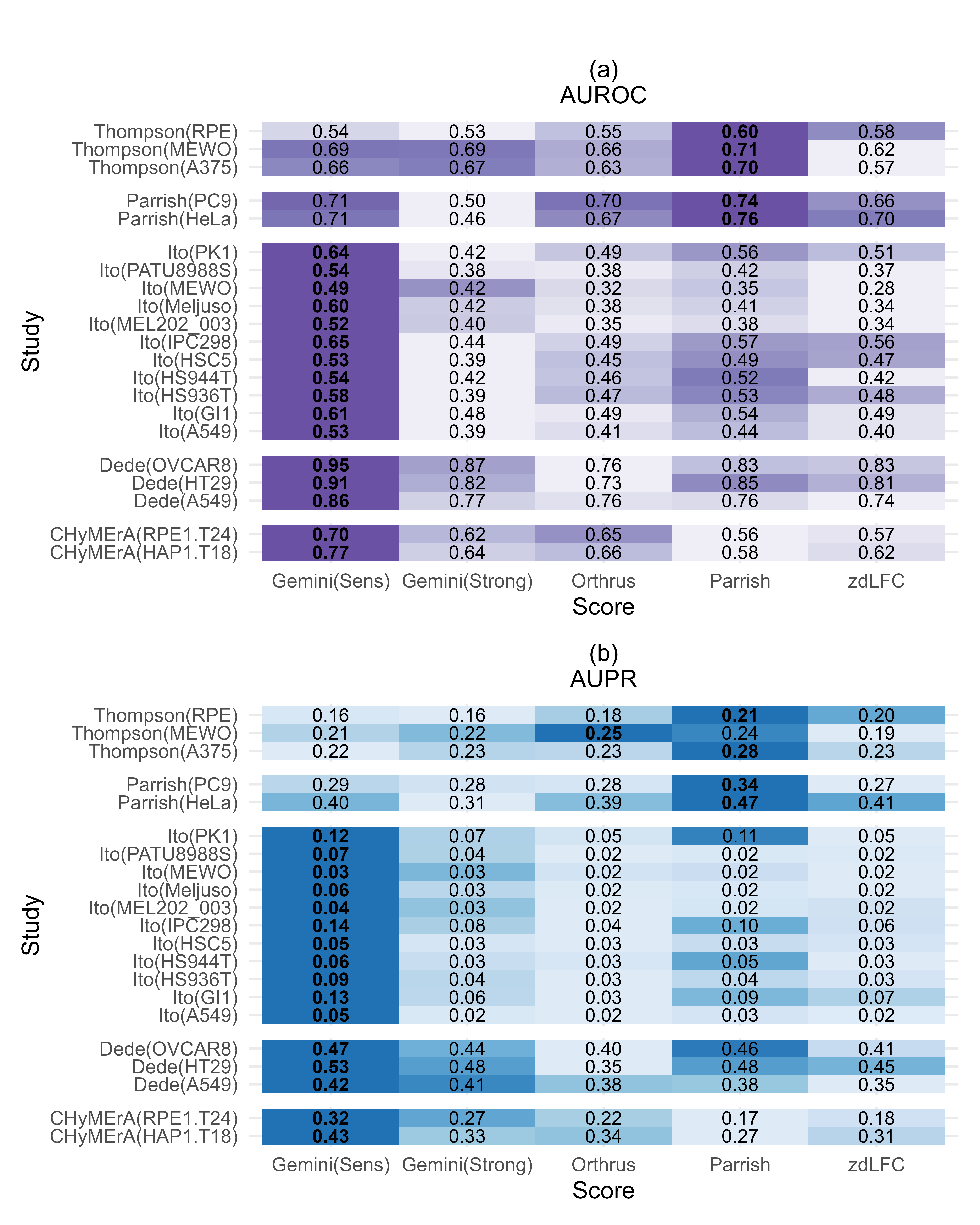


**Figure S4.** Evaluating scoring systems using the Köferle benchmark. (a) AUROC and (b) AUPR across individual cell lines of each study using Köferle benchmark. The colour gradients are study specific. As each study has different baseline precision rates, this graph should be used to compare scoring methods across an individual study rather than across studies.


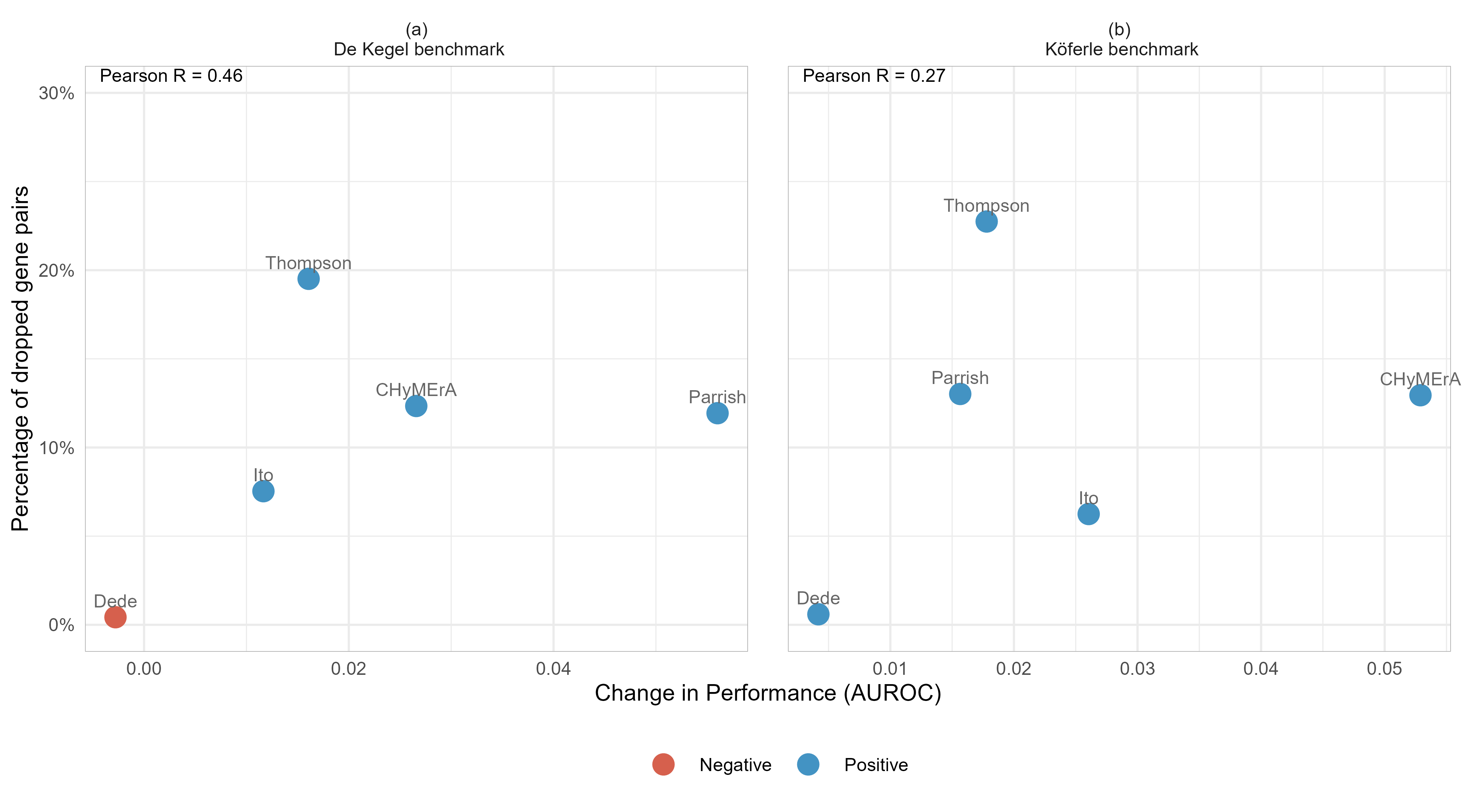


**Figure S5.** Relationship between number of essential genes in the screen design and improvement of Gemini-Sensitive score performance with the application of default filter. X-axis shows the change in AUROC when default filtering step of Gemini is applied, and y-axis shows the percentage of gene pairs with at least one essential gene dropped by the Gemini default filter. A positive (blue) change shows improvement in performance.

**
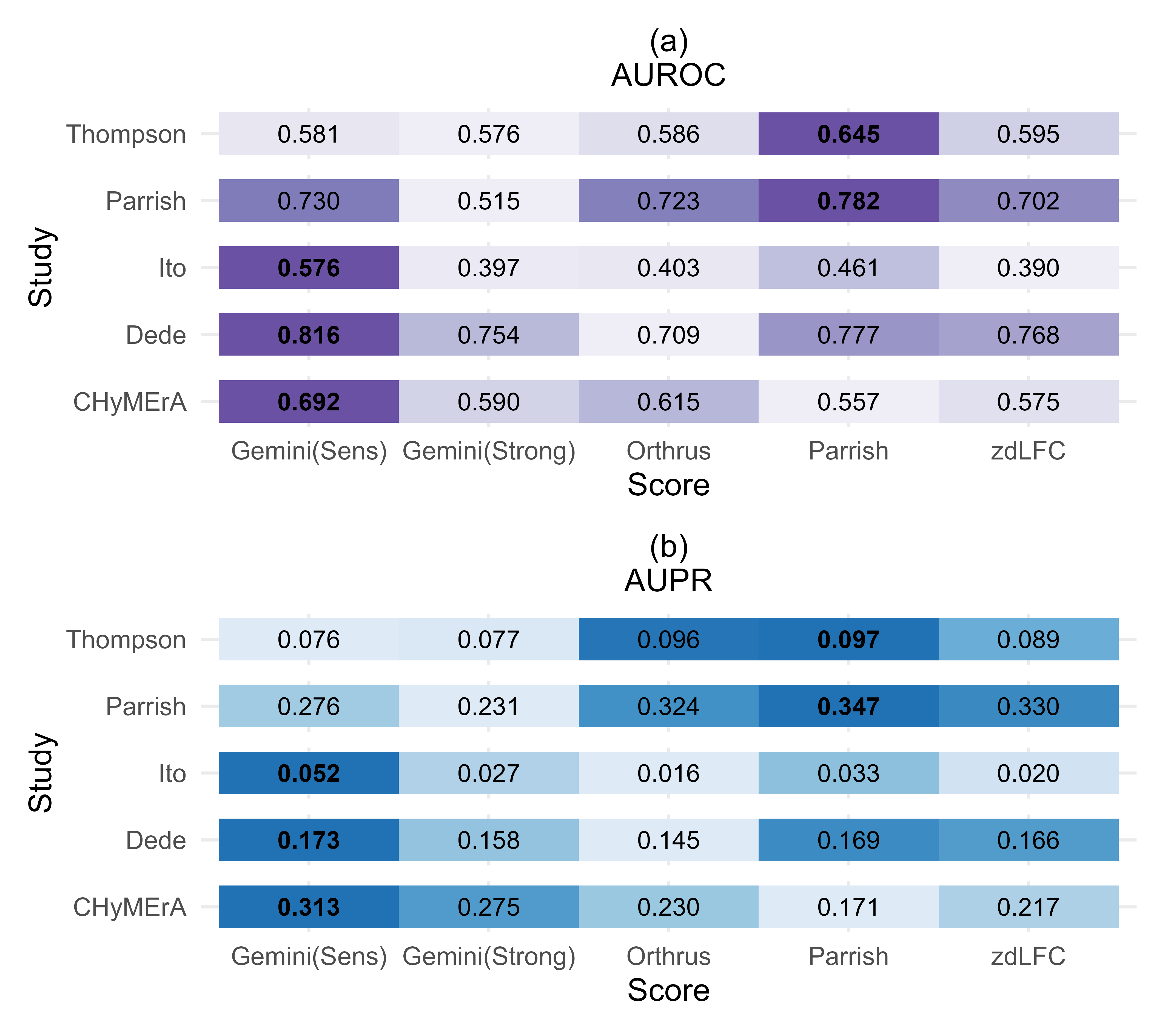
**

**Figure S6. Evaluation of scoring systems using the BaCoN benchmark.** (a) AUPR and (b) AUROC of each study with all cell lines combined. The colour gradients are study specific. The colour represents the normalized AUROC/AUPR for each study, with the darkest shade of purple/blue indicating the highest AUROC/AUPR within the corresponding study. As each study has different baseline precision rates, this graph should be used to compare scoring methods across an individual study rather than across studies.

**
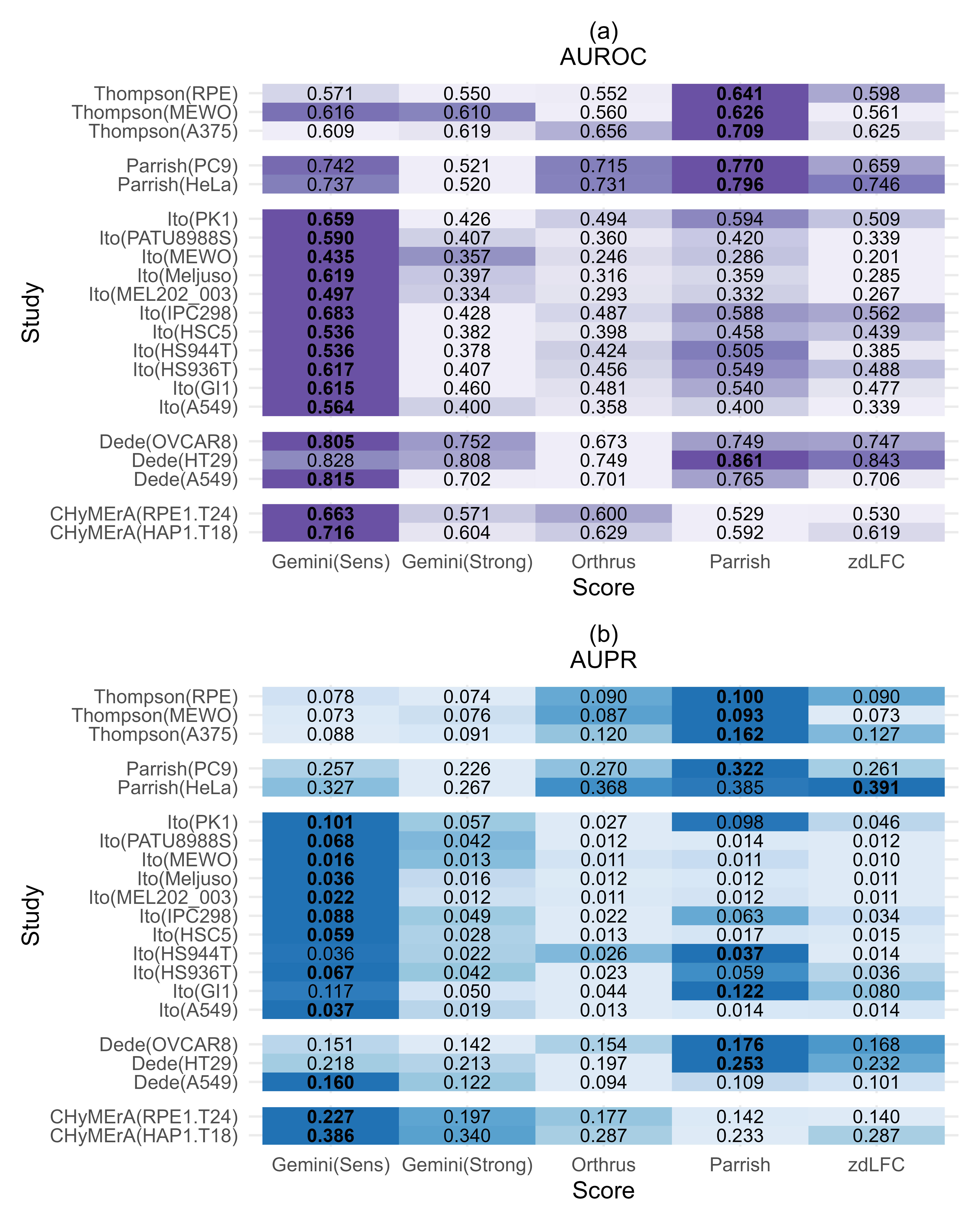
**

**Figure S7.** Evaluating scoring systems using the BaCoN benchmark. (a) AUROC and (b) AUPR across individual cell lines of each study using BaCoN benchmark. The colour gradients are study specific. As each study has different baseline precision rates, this graph should be used to compare scoring methods across an individual study rather than across studies.
